# Harnessing diversity and antagonism within the pig skin microbiota to identify novel mediators of colonization resistance to methicillin-resistant *Staphylococcus aureus*

**DOI:** 10.1128/msphere.00177-23

**Published:** 2023-07-05

**Authors:** Monica Wei, Laurice Flowers, Simon A. B. Knight, Qi Zheng, Sofia Murga-Garrido, Aayushi Uberoi, Jamie Ting-Chun Pan, Jasmine Walsh, Erin Schroeder, Emily W. Chu, Amy Campbell, Daniel Shin, Charles W. Bradley, Raimon Duran-Struuck, Elizabeth A. Grice

**Affiliations:** 1 Department of Dermatology & Microbiology, Perelman School of Medicine, University of Pennsylvania, Philadelphia, Pennsylvania, USA; 2 Department of Pathobiology, School of Veterinary Medicine, University of Pennsylvania, Philadelphia, Pennsylvania, USA; University of Wisconsin-Madison, Madison, Wisconsin, USA

**Keywords:** skin, skin microbiome, microbe–microbe interactions, *Staphylococcus aureus*, porcine skin, colonization resistance, antagonism

## Abstract

The microbiota mediate multiple aspects of skin barrier function, including colonization resistance to pathogens such as *Staphylococcus aureus*. The endogenous skin microbiota limits *S. aureus* colonization via competition and direct inhibition. Novel mechanisms of colonization resistance are promising therapeutic targets for drug-resistant infections, such as those caused by methicillin-resistant *S. aureus* (MRSA). Here, we developed and characterized a swine model of topical microbiome perturbation and MRSA colonization. As in other model systems, topical antimicrobial treatment had a little discernable effect on community diversity though the overall microbial load was sensitive to multiple types of intervention, including swabbing. In parallel, we established a porcine skin culture collection and screened 7,700 isolates for MRSA inhibition. Using genomic and phenotypic criteria, we curated three isolates to investigate whether prophylactic colonization would inhibit MRSA colonization *in vivo*. The three-member consortium together, but not individually, provided protection against MRSA colonization, suggesting cooperation and/or synergy among the strains. Inhibitory isolates were represented across all major phyla of the pig skin microbiota and did not have a strong preference for inhibiting closely related species, suggesting that relatedness is not a condition of antagonism. These findings reveal the porcine skin as an underexplored reservoir of skin commensal species with the potential to prevent MRSA colonization and infection.

**IMPORTANCE:**

The skin microbiota is protective against pathogens or opportunists such as *S. aureus*, the most common cause of skin and soft tissue infections. *S. aureus* can colonize normal skin and nasal passages, and colonization is a risk factor for infection, especially on breach of the skin barrier. Here, we established a pig model to study the competitive mechanisms of the skin microbiota and their role in preventing colonization by MRSA. This drug-resistant strain is also a livestock pathogen, and swine herds can be reservoirs of MRSA carriage. From 7,700 cultured skin isolates, we identified 37 unique species across three phyla that inhibited MRSA. A synthetic community of three inhibitory isolates provided protection together, but not individually, *in vivo* in a murine model of MRSA colonization. These findings suggest that antagonism is widespread in the pig skin microbiota, and these competitive interactions may be exploited to prevent MRSA colonization.

## INTRODUCTION

*Staphylococcus aureus* is a major cutaneous pathogen that also commonly asymptomatically colonizes the nares and skin of healthy adults (1). Colonization with *S. aureus* is a predisposing risk factor for infection and associated pathology (2, 3) and can precede a variety of different infections, ranging from local benign dermatopathologies (impetigo, folliculitis) to life-threatening abscesses, cellulitis, pneumonia, bone infection, and sepsis (4). Of particular concern, methicillin-resistant *S. aureus* (MRSA) infections have increased markedly over the past 20 years and are associated with significant excess morbidity, mortality, and cost (5). While *S. aureus* can colonize nares and skin asymptomatically, the factors that promote this pathogen’s shift from colonization to infection remain poorly understood.

*S. aureus* has also emerged as an important livestock pathogen and causes infections in economically important animals such as sheep, goats, pigs, cows, rabbits, and chickens (6). Livestock herds may act as a reservoir of newly virulent strains that cause infection in humans (7). Pigs, in particular, have been implicated as a reservoir of MRSA and an incubator for antimicrobial resistance, due in part to the common practice of feeding subtherapeutic antibiotics to promote growth (8 - 10). *S. aureus* strains that are pathogenic in livestock, specifically sequence type 398 (ST398; LA-MRSA), most likely originated in humans and later acquired antibiotic resistance cassettes after jumping to livestock hosts (11, 12). As antimicrobial resistance continues to deplete our therapeutic toolbox, there is an urgent need for novel treatment strategies, including those that are adaptable for human and livestock applications.

One factor that limits pathogen access and invasion is the skin’s endogenous microbiota, through a mechanism known as colonization resistance (13). Previous work has shown that disruption of the skin microbiota with broad-spectrum antimicrobials promotes colonization by *S. aureus* (14). Competitive interactions that limit *S. aureus* skin colonization and pathogenicity are common among the coagulase-negative staphylococci (CoNS) species. For example, *Staphylococcus hominis* and *Staphylococcus lugdunensis* produce novel antibiotics that kill *S. aureus (*15, 16). Interference with quorum sensing is another mechanism of competitive exclusion and is used by the CoNS species *Staphylococcus caprae* to limit bacterial growth and fitness of *S. aureus* (17). Additionally, skin commensals can stimulate and cooperate with host immune responses, thus playing an indirect role in excluding pathogens like *S. aureus*. Community-wide, these competitive interactions may in part explain how the commensal microbiota can exclude or counteract pathogens such as *S. aureus*.

Previous studies to characterize competitive interactions on the skin have largely focused on CoNS, which are prominent members of the skin commensal microbiota as established by culture-independent and -dependent studies. The bias toward CoNS species could reflect their adaptation and competitive advantage on the skin. It has also been postulated that competition is more common among closely related species (18). However, this tendency could also reflect cultivation bias since CoNS are readily cultured from the skin under the same conditions as *S. aureus* and MRSA. Recent studies have demonstrated that non-CoNS members of the skin microbiota also produce antibiotics, including *Cutibacterium acnes* (19). The extent to which skin commensals produce inhibitory molecules as means of competition is unknown, as is the link between phylogenetic relatedness and inhibitory capacity.

Model systems to isolate and study the skin microbiota often rely on murine models. Fundamental differences in murine skin compared to human skin include morphological and histological differences such as thickness, the density of pilosebaceous units and other appendages such as sweat glands, and even distinct pathways and cell types involved in repair processes (20 - 22). Furthermore, many *S. aureus* virulence factors are attenuated in mice (23). On the other hand, porcine skin displays similar morphology and histology to human skin and has similar density of appendages (24) and may represent a more suitable host for modeling skin microbial communities and MRSA colonization. *S. aureus* is also a natural pathogen of pigs, likely owing to similarities to human skin and nares. Because microbially produced antibiotics are often specific to the range of species and environmental conditions likely to be encountered (25), the pig skin microbiota may also harbor novel bacterial determinants and mechanisms of microbial antagonism. Inhibitory mechanisms applicable to livestock populations would have important implications for the veterinary control of *S. aureus*, in addition to the implications for human health and disease.

Here, we developed and characterized a pig skin model system to study microbial community perturbations and colonization resistance to MRSA. We first characterized the microbial diversity of porcine skin using cultures and 16S rRNA gene sequencing. We tested the resilience of the skin microbiota to pulse disturbances of various topical antimicrobial drugs and then examined community resistance to MRSA colonization. This experimental design together with a high-throughput *in vitro* inhibition screen led to the identification of 37 unique species that inhibited MRSA. We colonized murine skin with three candidate inhibitors to test whether prophylactic colonization with pig commensal isolates also inhibited MRSA *in vivo*. We further investigated the relationship between phylogenetic relatedness and antagonism to address whether closely related (e.g., *Staphylococcus*) species were more likely to display inhibition.

## RESULTS

### Bacterial composition of the dorsal skin microbiota of swine

**Fig 1:**
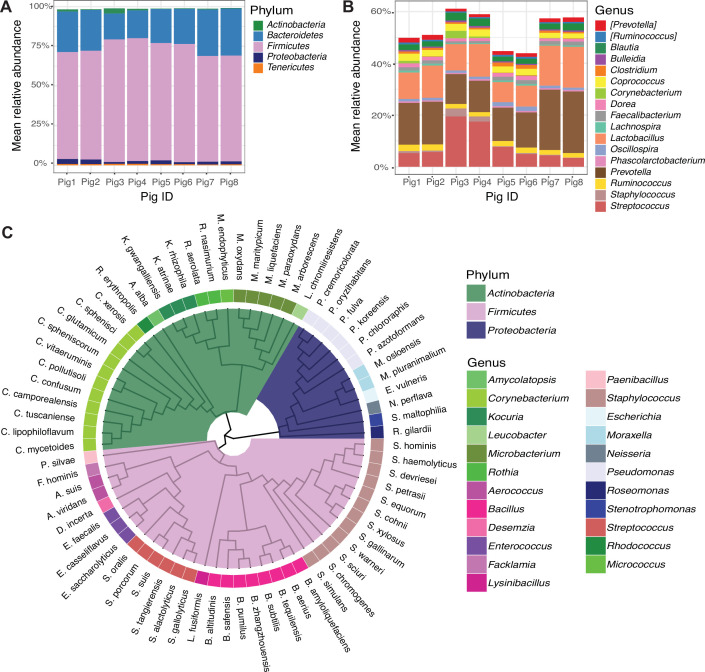
The culture-independent and -dependent microbiota of porcine skin. Mean relative abundance of top (A) phyla and (B) genera present in dorsal pig skin microbiota by 16S rRNA gene sequencing. Each bar represents an individual pig. (C) Cladogram of bacterial isolates cultured from pig skin. Phylogeny was constructed from representative 16S rRNA gene sequences curated from the Silva database (see Materials and Methods).

We first characterized the baseline, unperturbed skin microbiota of Yucatan pig dorsal skin by analyzing 16S rRNA gene sequences. We demarcated 10 distinct squares on the dorsum each measuring ~40 cm^2^ (Fig. S1) and collected swabs from each square to sample the baseline skin microbiota by amplicon sequencing of 16S rRNA genes. Thus, 10 replicates per pig (*n* = 8 pigs total) were collected, corresponding to each square, for a total of 80 swab samples at baseline. We observed at the phylum level similarities with the human bacterial skin microbiota, including a predominance of Firmicutes in the community (Fig. 1A). At the genus level, the most abundant taxa were *Streptococcus*, *Prevotella*, and *Lactobacillus* (Fig. 1B). Genera that predominate on human skin were also found on porcine skin, albeit at lower relative abundances, including *Staphylococcus* and *Corynebacterium*. The effect of cohousing of the pigs was also apparent, as our studies were conducted over four experiments of two pigs each, and batch effects are visible across paired individuals.

In parallel with culture-independent 16S rRNA amplicon sequencing, we characterized the culturable microbiota by plating skin swabs on blood agar under aerobic conditions. We identified 84 unique species or presumptive species using matrix-assisted laser desorption/ionization time-of-flight (MALDI-TOF) mass spectrometry and full-length sequencing of the 16S rRNA gene for identification (Table S1). As observed in the phylum-level culture-independent data, the top phylum recovered was Firmicutes, but we also cultured a substantial proportion of isolates that were identified as Actinobacteria and Proteobacteria (Fig. 1C). We did not recover isolates from the phyla Bacteroidetes or Tenericutes (Mycoplasmatota), which were identified in the 16S amplicon data, nor did we recover all of the genera identified. Obligate anaerobes, such as Bacteroidetes, *Prevotella*, *Clostridium*, and *Lachnospiraceae*, were absent from cultured isolates. This is not surprising since isolates were cultured under aerobic conditions but also highlights the limitations of culture-based approaches for comprehensive microbial community profiling. Genera and species known to inhabit human skin were more readily recovered, including 12 species of coagulase-negative staphylococci, *Staphylococcus hominis*, *Staphylococcus haemolyticus*, *Staphylococcus warneri*, *Staphylococcus cohnii*, and *Staphylococcus simulans*. Eleven species of *Corynebacterium* were isolated including the food-industry staple *Corynebacterium glutamicum*, the zoonotic pathogen *Corynebacterium xerosis*, and human skin–associated species such as *Corynebacterium lipophiloflavum* and *Corynebacterium mycetoides*. Overall, these results highlight some similarities between porcine and human skin microbiota at the genus level but also suggest a distinct cutaneous ecosystem at the species level.

### Broad-spectrum antibiotic and antiseptic treatment alter MRSA colonization on porcine skin

**Fig 2:**
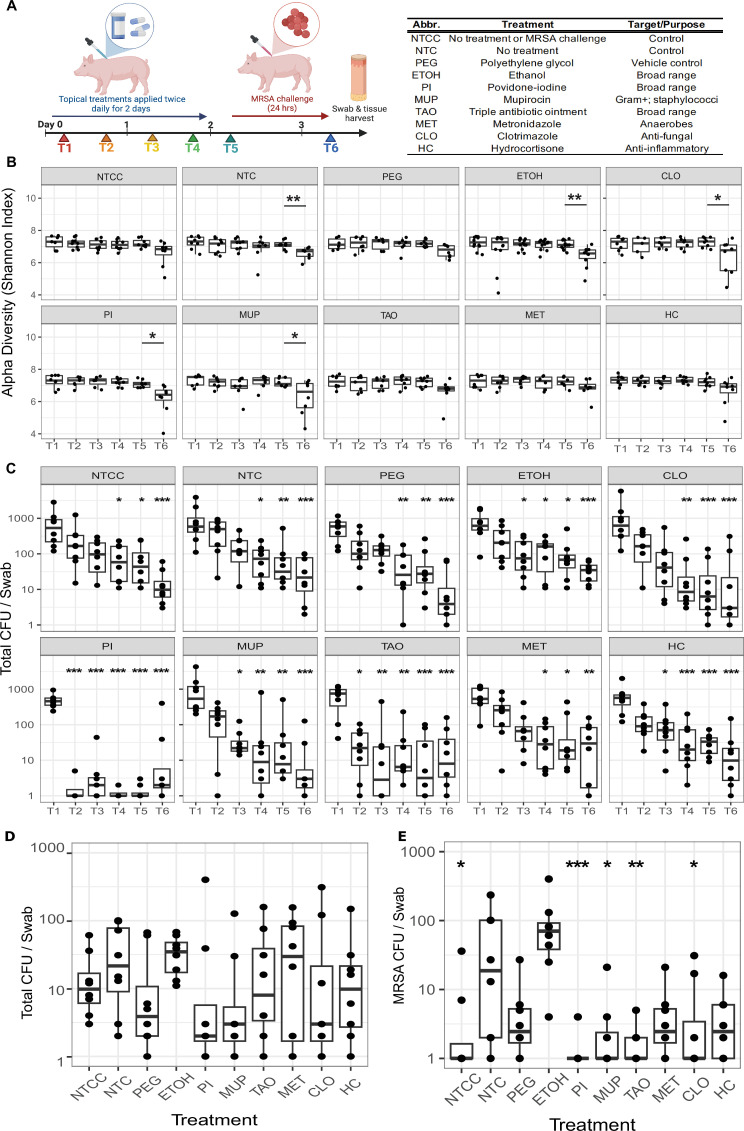
Skin microbiota dynamics during antimicrobial treatment and MRSA challenge. (**A**) Experimental design for topical treatment and MRSA colonization. The accompanying table lists the different treatments and controls topically applied to the skin. Swabs were collected at each time point (T1–T6) for 16S rRNA amplicon sequencing and CFU quantification. After swab collection at T1–T4, treatments were applied to the skin. After swab collection at T5, MRSA was applied to the skin. At T6, 24 hours later, final swabs and tissues were collected. (**B**) Shannon diversity index (*y*-axes) represented over time, T1–T6 (*x*-axes). Each panel represents a different treatment, as indicated in the gray heading. To determine how treatments impacted diversity, statistical testing was performed between T1 and T2, T3, T4, and T5. To determine how MRSA treatment impacted diversity, statistical testing was performed between T5 and T6 (Wilcoxon test); (**C**) total CFU recovered (*y*-axes) over time, T1–T6 (*x*-axes). Each panel represents a different treatment, as indicated in the gray heading. Statistical testing was performed between T1 and all other time points for each treatment group individually using a two-way analysis of variance (ANOVA) with Dunnett’s *post hoc* analysis and Bonferroni correction; (**D**) total CFU recovered (*y*-axis) at the final T6 time point (treatment groups compared to NTC, ANOVA with Dunnett’s *post hoc* analysis); (**E**) CFU of MRSA (*y*-axis) recovered at T6 time point following the 24-hour challenge for each treatment (*x*-axis) (treatment groups compared to NTC control, ANOVA with Dunnett’s *post hoc* analysis A pseudocount of +1 was added to all CFU counts to accommodate the log scale. CFUs were log-transformed before statistical analysis. *P < 0.05, **P < 0.01, ***P < 0.001.

We previously observed in hairless (SKH-1 elite) mice that topical application of ethanol promoted *S. aureus* colonization (14). We performed similar experiments using the swine model to test the effect of eight topical perturbations on skin microbiota diversity and MRSA colonization. After collecting baseline microbiota specimens for characterization as described above, the 10 demarcated skin patches were treated twice daily for 2 days with topical interventions consisting of antiseptics (ethanol [ETOH], povidone-iodine [PI]), an antifungal (clotrimazole [CLO]), antibiotics (metronidazole [MET], mupirocin [MUP], triple antibiotic ointment [TAO]), and an anti-inflammatory (hydrocortisone [HC]) (Fig. 2A). All ointments were prepared in a polyethylene glycol (PEG) vehicle, thus a PEG control was included in addition to two untreated controls (NTC and NTCC). To functionally characterize the skin microbiome configurations that result from topical pulse disturbance, each skin patch was challenged with 1 × 10^8^ CFU MRSA (USA300 Rosenbach strain) at time point 5 (T5) (Fig. 2A). Swabs were collected for both culture-dependent and -independent profiling, twice daily, in the morning and evening, approximately 8 hours apart.

We first examined how treatment altered the diversity of the skin microbiota and found that the topical pulse disturbances had little discernable effect, as measured by the Shannon diversity index (Fig. 2B). MRSA challenge resulted in a significant decrease in community diversity when the skin was pre-treated with ETOH, PI, HC, as well as the vehicle PEG, as estimated by the Shannon diversity index (Fig. 2B). The depletion in alpha diversity was consistent with an increase in the relative abundance of *Staphylococcus* by 16S rRNA amplicon sequencing at time point 6 (Fig. S2). However, quantification of cultured CFU indicated a reduction of bacterial load on the skin (Fig. 2C). In all treatment groups and the control group, there was a significant depletion of bacterial CFUs at later time points; this likely reflects the effect of repeated swabbing over the same area. To account for this effect and further investigate the differences in group-level trajectories over time, we fitted the natural log values of the total CFU counts using a random-effect generalized least squares (GLS) model with linear splines. A two-spline model with a knot at the second time point sufficiently described the trajectories by group (Fig. S3). Both PI (*P* < 0.001) and TAO (*P* = 0.003) had significantly different trajectories compared to NTCC, with a substantial initial decrease in log counts. This result suggests that PI and TAO caused an additional initial decrease in total bacterial load in addition to the effect seen from swabbing alone. CFUs of MRSA and total bacteria were quantified 24 hours after the MRSA challenge. Quantification of total CFU (Fig. 2D) and MRSA CFU (Fig. 2E) at the final time point indicated that the MRSA challenge had no discernable effect on total CFU in any treatment group. As expected, MRSA CFU was significantly decreased in untreated/unchallenged control. MRSA CFU was also significantly decreased in the PI, MUP, TAO, and CLO groups, suggesting that these treatments may be effective in preventing MRSA colonization in pigs. We did not observe significant effects when comparing the ratio of MRSA to total CFU across treatments (Fig. S4).

### Identification of phylogenetically diverse pig commensal isolates with anti-MRSA inhibitory activity

**Fig 3:**
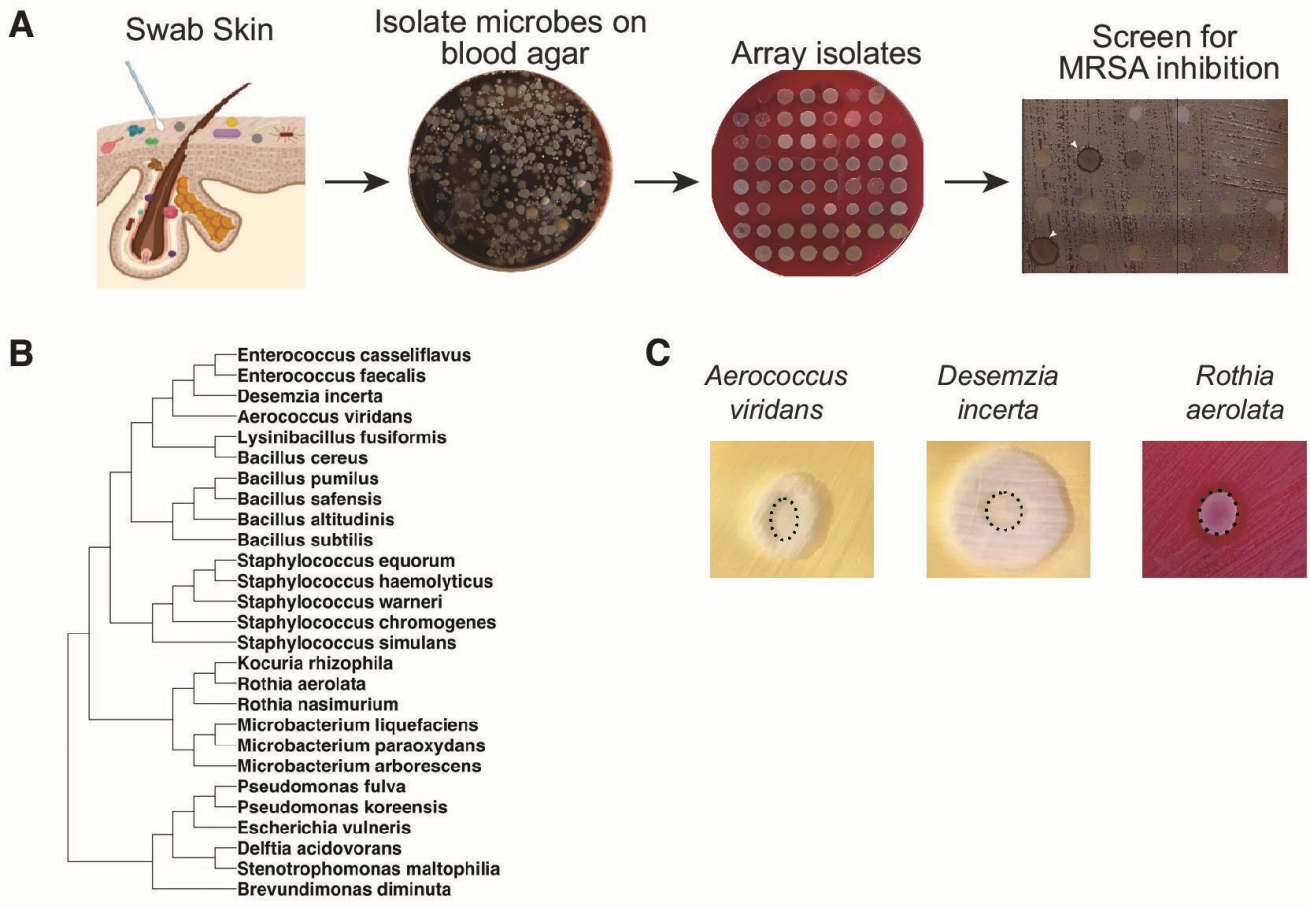
High-throughput screening of pig skin commensal library identifies novel inhibitors of MRSA. (**A**) Strategy for isolating and screening pig skin isolates for MRSA inhibitory activity; (**B**) cladogram of bacterial inhibitors of MRSA isolated from porcine skin; phylogeny was constructed from representative 16S rRNA sequences from the Silva database; (**C**) photographs of MRSA inhibition for each of the three selected isolates. USA300 strain MRSA, *Aerococcus viridans*, *Desemzia incerta*, and *Rothia aerolata* were grown overnight in BHI + 0.8% Tween 80 media. MRSA was diluted to OD_600_ of 0.1 and spread on BHI-T agar (for *A. viridans* and *D. incerta*) or tryptic soy agar (TSA) + 5% blood agar (for *R. aerolata*). Pig skin isolates equivalent to an OD_600_ of 2.0 were spotted on the MRSA lawn. The border between the spot colony and the zone of inhibition is demarcated in dotted lines.

A total of 7,700 cultured skin isolates were spotted on a lawn of MRSA grown on blood agar to screen for inhibitory activity (Fig. 3A). Of these isolates, 363 exhibited clearing, or a zone of inhibition (ZOI) surrounding the spot on the MRSA lawn. Identification of this group of isolates by MALDI-TOF mass spectrometry or sequencing of the 16S rRNA gene indicated that it comprised 37 unique species (Fig. 3B; Table S1). Fifty-four isolates were not identified, and 58 were assigned at the genus level. *Aerococcus viridans* was the most common MRSA-inhibiting isolate (40 isolates of the 363) followed by *S. warneri* (26 isolates) and *Bacillus pumilus* (19 isolates). Six different species of *Staphylococcus* were identified all of which were coagulase-negative. Twenty-five of the isolates identified at the species level were represented by a single isolate, including a relatively undescribed bacterium *Desemzia incerta*. Not all inhibitory isolates were bacteria, and since fungi comprise a significant component of both the human and pig skin microbiota (26, 27), we included recovered isolates identified as fungi in our screen. Single isolates of *Trichosporon asahii* and *Cryptococcus magnus* as well as three isolates of *Candida guilliermondii* were captured as MRSA inhibitors.

### A consortium of inhibitory bacteria provides protection against MRSA colonization *in vivo*

**Fig 4:**
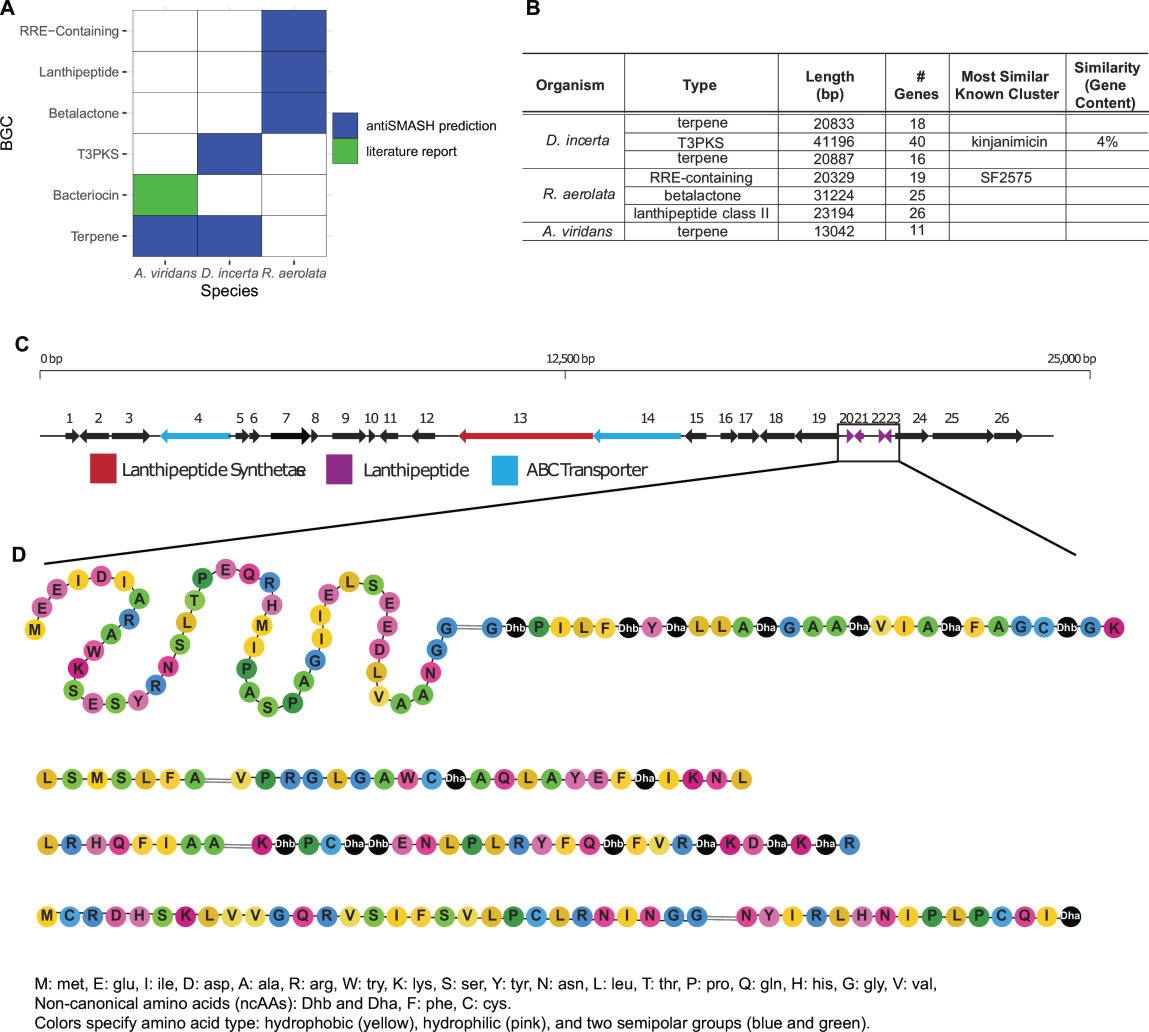
Genomic analysis of select isolates indicates non-overlapping mechanisms of inhibition. (**A**) Biosynthetic gene cluster (BGC) profiles based on genomic data (blue) or previous study (green) for three pig isolates; (**B**) summary table of antiSMASH BGC prediction in each isolate; (**C**) gene map of prediction lanthipeptide BGC in *Rothia aerolata*; (**D**) predicted lanthipeptide structures of the lanthipeptide biosynthetic product. – indicates cleavage site. Dha, dehydroalanine; Dhb, dehydrobutyrine; RRE, ribosomally synthesized post-translationally modified peptide (RiPP) recognition element; T3PKS, type 3 polyketide synthesis.

We further focused on three inhibitory species in particular, *A. viridans*, *D. incerta*, and *R. aerolata* (Fig. 4A). These were selected because they are phylogenetically diverse, inhibited MRSA under distinct conditions, and were predicted to use different mechanisms of inhibition. *A. viridans* and *D. incerta* exhibited the strongest inhibition of MRSA growth at RT on media in the absence of blood. Higher temperatures progressively decreased the MRSA inhibitory activity of these two species, and the presence of blood decreased the MRSA inhibitory activity at all temperatures (Fig S5). *R. aerolata* exhibited a more refractory MRSA inhibition; however, its growth was robust on a lawn of MRSA at 37°C in the presence of blood, compared to *A. viridans* and *D. incerta* (Fig. S5).

Genomic sequencing of the three isolates enabled the prediction of biosynthetic gene clusters (BGCs), comprising the genes needed to produce, process, and export small molecules and peptides that mediate interactions between species. Two different algorithms, deepBGC (28) (Fig. S6) and antiSMASH (29), were tested. We present antiSMASH results preferentially as it has a low false-positive rate and the ability to predict chemical structures (30). This analysis (antiSMASH v6) indicated non-overlapping sets of BCGs between the three isolates (Fig. 4A). *D. incerta* BGCs included a type 3 polyketide synthase (T3PKS) and a terpene. *D. incerta* has previously been associated with extracellular enzymatic activities that promote efficient composting (31, 32), as well as the production of a lactonase enzyme that degrades homoserine lactone molecules, effectors of quorum sensing expressed by Gram-negative bacteria (33). *A. viridans* BGCs included a terpene. Prior studies in *A. viridans* have also identified and isolated a bacteriocin, viridicin, that inhibited Gram-negative and -positive pathogens (34). *R. aerolata* contained BGCs predicted to encode a lanthipeptide, a betalactone, and an RRE (ribosomally synthesized post-translationally modified peptide (RiPP) recognition element)-containing cluster though no prior reports were found of its inhibitory properties (Fig. 4B).

We found the lanthipeptide BGC in *R. aerolata* particularly promising, as previously characterized lanthipeptides have a high rate of confirmed antimicrobial activity (35). The lanthipeptide BGC is predicted to contain a lanthipeptide synthetase gene, multiple transport genes, and several short peptide sequences that could potentially be modified to an active lanthipeptide (Fig. 4C). This gene cluster, therefore, contains all the elements required for lanthipeptide synthesis (35). We used the antiSMASH lanthipeptide module to model the post-translationally modified forms of the lanthipeptide product including cleavage sites and the presence of non-canonical amino acids dehydroalanine and dehydrobutyrine. Predicted peptide sequences of four lanthipeptide products from the four lanthipeptide genes found in the gene cluster are shown in Fig. 4D.

We further found that *A. viridans* and *D. incerta* additionally inhibited *Pseudomonas aeruginosa*, and *D. incerta* also inhibited *Streptococcus pyogenes* (Fig. S7). Taken together, this evidence suggested the three isolates are likely to employ non-overlapping mechanisms of inhibition.

**Fig 5:**
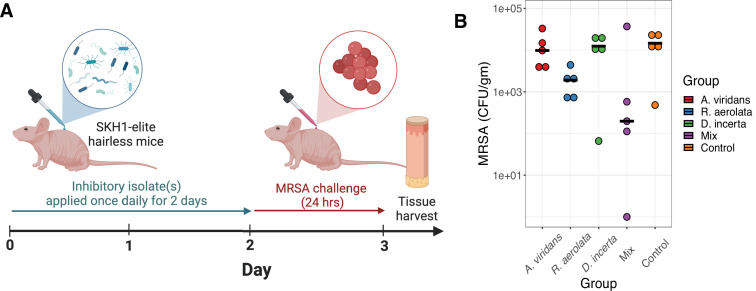
A defined consortium of pig skin isolates reduces MRSA colonization on murine skin. (**A**) Schematic of experimental design for murine colonization experiments; (**B**) CFU of MRSA recovered (*y*-axis) when the skin is pre-colonized with the indicated isolate, a mix of all 3, or control. *N* = 5 mice per group. Bars indicate the median for each treatment group. Statistical testing was performed comparing the treatment group to the control using ANOVA with Tukey’s post hoc analysis. A pseudocount of +1 was added to all CFUs to accommodate the log scale. Log-transformed values were used for statistical analysis.

While the three porcine skin isolates we selected to inhibit MRSA *in vitro*, it was uncertain whether these isolates would provide the same protection *in vivo*. Furthermore, since the mechanisms of inhibition were likely different, we hypothesized that using the three isolates in a mix (3-Mix) would provide more protection than monocolonization with a single inhibitory isolate. To test this hypothesis, we returned to a hairless mouse model we previously used in the studies of skin colonization resistance (14). SKH-1 elite mice were colonized prophylactically with each isolate or the 3-Mix daily for 2 days (Fig. 5A). On the third day, the skin was challenged with MRSA (1 × 10^8^). After 24 hours, the skin was collected and processed to quantify MRSA CFU and to calculate colonization efficiency. We performed a pilot study with 4.5 × 10^7^ inoculum of each pig isolate and found that individually, no single isolate was consistently effective at disrupting MRSA colonization (Fig S8). However, the 3-Mix blocked MRSA colonization in three of the four mice, to a median 733 CFU per gram of skin, compared to a median 1.06 × 10^4^ CFU per gram of skin in the control. Because there was a large amount of variability between mice, we repeated the experiment with a higher inoculum of the pig commensal isolates (1 × 10^8^ each isolate). Similar to the pilot experiment, four of the five mice treated with the 3-Mix had ~2-log decrease in MRSA colonization compared to the controls (median = 1.96 × 10^2^ and 1.46 × 10^4^ CFU, respectively) (Fig. 5B). While the differences we observed were not statistically significant (analysis of variance [ANOVA], *P* = 0.07), the consistency of the findings over independent experiments suggests that the 3-Mix is more effective than each component individually in providing protection against MRSA colonization in this model.

To test whether the individual species’ inability to resist MRSA colonization *in vivo* was related to their persistence on skin, we performed an additional experiment testing colonization efficiency of hairless mice with each of our three inhibitory isolates separately. After 2 days of successive colonization with 10^8^ CFU of each isolate, we plated bacteria from 6-mm punch biopsies and analyzed 10 bacterial colonies per mouse by MALDI-TOF, selecting specifically for small white colonies which resemble the isolates of interest. *A. viridans* accounted for 14/30 (47%) colonies, *R. aerolata* accounted for 17/30 (56%) colonies, and *D. incerta* accounted for 0/15 colonies selected from colonized mice. In the tryptic soy broth (TSB) control mice, 0/30 colonies were identified as one of the species of interest. This suggests that there are differences in colonization efficiency of the three pig-derived isolates on murine skin; however, this colonization efficiency did not correlate well with the MRSA resistance phenotype we observed, as monocolonization with the three isolates seemed to have a similar effect on MRSA colonization resistance, with *D. incerta* trending toward the highest level of MRSA reduction.

### Phylogenetic distance is not strongly correlated with antagonism

**Fig 6:**
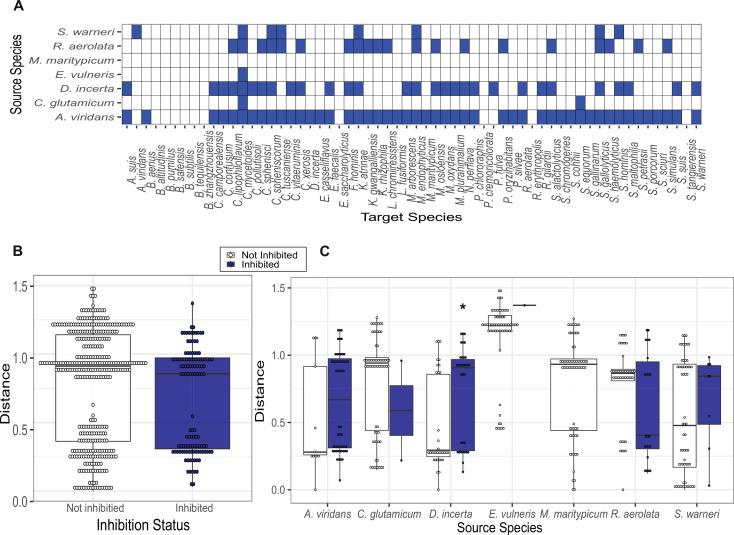
Phylogenetic distance is not related to inhibition status. (**A**) Heatmap showing inhibitory interactions (blue) between seven “Source” isolates and 60 “Target” isolates from the pig commensal culture collection. (**B**) Patristic distance between bacterial isolate pairs that showed an inhibitory interaction (blue) versus a non-inhibitory interaction (white) on agar diffusion assay (Wilcoxon test). (**C**) Patristic distance between bacterial isolate pairs that showed an inhibitory interaction versus non-inhibitory interaction, separated by “Source” species (inhibited versus non-inhibited for each group; Wilcoxon test with Bonferroni correction).

Studies of environmental microbial communities have demonstrated a correlation between phylogenetic distance and microbial antagonism (18). In line with this finding, previous studies of competitive microbial interactions on the skin have largely identified CoNS as inhibitors of *S. aureus* (15, 16). We were, therefore, surprised to find that only 6/37 (16.2%) of MRSA inhibitors that we isolated from porcine skin were staphylococcal species, suggesting that phylogenetically distant inhibitors of *S. aureus* may be common in some microbial communities. To test whether bacteria from porcine skin showed a preference for inhibiting phylogenetically similar species, we selected seven isolates (“Source” species) for further characterization of inhibition patterns. Five isolates—*D. incerta*, *A. viridans*, *R. aerolata*, *Escherichia vulneris*, and *S. warneri—*were inhibitors of MRSA, while two isolates*—C. glutamicum* and *Microbacterium maritypicum* were not inhibitors of MRSA. Using the agar diffusion assay described above, we performed pairwise inhibition testing of these seven source species against a panel of 60 “target” species, which were unique and phylogenetically diverse isolates that were selected from the 72 unique species cultured from pig skin and shown in Fig. 1C. We tested whether the source species inhibited the target species on two growth media (BHI-T agar and blood agar) and two temperature conditions (37°C and RT, ~22°C–23°C). An interaction was considered inhibitory if the target species was inhibited in at least one growth condition. A total of 1,553 assays were performed across all growth conditions, which were consolidated into 420 pairwise interactions (Fig. 6A). For each pairwise interaction, we estimated phylogenetic distance based on a multiple sequence alignment of representative, publicly available 16S rRNA gene sequences. The longest cophenetic longest distance was 1.26 (between *E. vulneris* and *Streptococcus gallolyticus*, members of two different phyla), while the shortest cophenetic distance was 0.001 (between *B. pumilus* and *Bacillus zhangzhouensis*, two species in the same genus).

Across all interactions, we found a smaller mean cophenetic distance between inhibitory pairs compared to non-inhibitory pairs; this difference was not statistically significant but strongly trending (Fig. 6B; *P* = 0.055). This finding is consistent with other studies demonstrating a higher probability of bacterial antagonism between phylogenetically related species. However, when interactions were separated by source species, this trend was not reproduced. Six out of seven species showed no statistically significant relationship between phylogenetic distance and inhibitory interaction. *D. incerta* showed a higher mean phylogenetic distance between inhibited species compared to non-inhibited species (Fig. 6C; *P* = 0.005). Thus, while a relationship between phylogenetic distance and bacterial inhibition seems to exist at the broader community level, individual bacterial species exhibited a diverse range of behaviors.

## DISCUSSION

Here, we developed and employed a porcine skin microbiome model to identify novel bacterial determinants of colonization resistance to the skin and soft tissue pathogen MRSA. Combining culture-dependent and -independent approaches, we find that the pig skin microbiota has some similarities to human skin communities, including a phylogenetic preference for Actinobacteria and Firmicutes. We cultured and screened 7,700 pig skin isolates and identified 363 with inhibitory activity against MRSA. The majority of these inhibitory isolates were non-staphylococcal species and could inhibit other bacteria across a broad phylogenetic range. Combining phenotypic and genomic analysis, we selected three distinct isolates to examine their capacity to inhibit MRSA *in vivo*. We found that the best protection was provided by a consortium of the three inhibitory isolates and that monocolonization with individual inhibitors was not as effective.

Our rationale for pursuing novel bacterial determinants of colonization resistance in the pig model is three-fold: (i) pig skin resembles human skin more than other commonly used model organisms; (ii) livestock, including swine, can be infected and act as reservoirs for *S. aureus* and MRSA; and (iii) the understudied ecosystem of the pig microbiome may contain novel mediators of colonization resistance that could be used to combat drug-resistant infections like MRSA. The pig model also has some disadvantages, such as the cost and labor associated with husbandry, and the unavailability of tools and reagents (e.g., antibodies) to study host-related factors. In this case, the utility of the pig model was most apparent in the initial screen to identify novel isolates that inhibit *S. aureus*.

Despite their utility in wound-healing studies, the pig skin microbiota is relatively underexplored. Here we profiled the dorsal pig skin microbiome using parallel cultures and 16S rRNA amplicon sequencing, at baseline and in response to topical perturbation. Previous reports of the culture-independent microbial communities of dorsal pig skin have observed similar types of microbiota as we report here, including representation primarily from the phyla Firmicutes, Actinobacteria, Bacteroidetes, and Proteobacteria (36). Slaughterhouse pig skin was enriched with *Aerococcus* compared to our research facility pigs but also revealed similar genera as we report including *Streptococcus*, *Staphylococcus*, *Corynebacterium*, and *Lactobacillus* (37). As observed previously (36), we detected comparable phyla and genera as those that reside on human skin, but divergence occurred at the species level. Sampling location may play a role in the divergence we observed, as dorsal skin was selected for our treatment experiments since it provides a large area of accessible skin of similar quality. As in humans, *Staphylococcus* and *Corynebacterium* spp. likely localize to moist, intertriginous regions of the pig, such as ear, axilla, or groin (37).

Previous to this work, the majority of skin microbial strains that have been investigated for *S. aureus* inhibition were other staphylococcal species (13). We speculated that this might reflect the ease of isolation and dominance in the human skin microbiota, rather than a phylogenetic bias of closely related species inhibiting each other. In line with previous studies from soil microbes, we found that the porcine skin isolates we examined were more closely related to species they inhibited than to non-inhibited species. However, the effect size between groups was very small (0.08, approximately the distance between two species in the same genus). Despite the limitation of only testing one strain per species, we found that bacterial species could inhibit other species across a broad phylogenetic range. We also identified many *S. aureus* inhibitors that were not *Staphylococcus* species. Furthermore, several inhibitory isolates were fungi, which are rich sources of metabolites with antimicrobial activity, including derivatives such as amoxicillin and cephalosporins (38). These findings suggest that genetically dissimilar skin microbiota still show a high rate of inhibition and should not be excluded from a search for inhibitory species or antimicrobial products.

Even though the 3-Mix of pig inhibitory isolates provided the most promising results for prophylactic colonization against MRSA, there was still a large amount of variability between mice that precluded statistical significance. The transplant of exogenous species to the skin can be hampered by the endogenous microbiota, in this case of hairless mice. Resource exclusion and/or antagonism by the microbiota, as well as host factors such as habitat filtering and immune mechanisms, are important factors that can influence the engraftment and function of a microbiota transplant (39). Thus, future studies will need to address these factors through an ecological framework, potentially by using germ-free and gnotobiotic mice, as well as investigating the impact of the inhibitory isolates on host immune response.

Previous studies have explored the potential of the human commensal microbiota, and strains within, to combat *S. aureus* colonization and infection (15 - 40 - 41 - 41). As antibiotic-resistant bacteria present an ever-increasing threat to our diminishing supply of antimicrobial drugs, alternative modes of therapy, such as bacteriotherapy, may offer promising opportunities to combat colonization and prevent infections. Our work demonstrates that similar to colonization resistance to vancomycin-resistant *Enterococcus* in the gut (42), a consortium of strains was required to provide protection against pathogen colonization. Other factors, such as the endogenous microbiota and the host may play a role in how transplanted communities function. Furthermore, leveraging the novel microbial–microbial interactions within underexplored microbial ecosystems that encounter similar threats (i.e., MRSA) may lead to novel antibiotic targets.

## MATERIALS AND METHODS

### Housing and care of pigs

Eleven 3- to 3½-month-old female Yucatan pigs (Sinclair Bio Resources LLC), weighing between 12 and 17 kg were housed in individual pens with unlimited access to food and water in a U.S. Department of Agriculture–approved ABSL2 facility. The pigs were acclimatized for 3 days and then were socialized for handling, by being fed cake-frosting and dog biscuits while being confined with a pig-board twice daily for 1 week. Three pigs were used in pilot experiments, and eight pigs were used for the main experiment. All experiments adhered to the regulations of the Animal Welfare Act and were approved by the University of Pennsylvania Institutional Animal Care and Use Committee (IACUC) Institutional Review Board no. 806346.

### Swab collection, application of topical ointments, and MRSA

The dorsal region of the pig was marked into 10 5- × 8-cm regions with permanent skin ink (Fig. S1). Four skin swabs from each region were taken, with each swab covering one-fourth of the region area (10 cm^2^), rotating swab collected in the specific patch at each sampling. Swabbing was performed using a sterile foam tip applicator (Puritan), moistened in phosphate-buffered saline (PBS) (Corning), and rubbed back and forth and crosswise with firm pressure for 10 seconds. The tip of one swab was broken off into a 2-mL tube (BioPur, Eppendorf) and snap frozen in dry ice and stored at −80°C for culture-independent analysis. The tip of the remaining three swabs was broken off into a 1.5-mL tube (BioPur) containing 0.3-mL sterile PBS to isolate microbiota by culture. This procedure was repeated at a total of six time points (T1–T6) which are detailed in Fig. 2A. T1 represents a baseline profile of the microbiota. After swab collection, interventions were added to the skin. Interventions were prepared by dissolving each compound (Fig. 2A) in 90% polyethylene glycol (PEG 3350) and applied with a sterile foam tip applicator to each marked region. This procedure was repeated at T2, T3, and T4, where the skin was swabbed followed by topical intervention. The time points were in the morning and evening, approximately 8 hours apart. At T5, the skin was swabbed and then challenged with MRSA (ATCC no. BAA-1717, Rosenbach strain of community-acquired USA300), grown at 37°C, 18 hours, 250 rpm in TSB (Becton Dickinson), and diluted to give 1 × 10^8^ CFU in 0.5-mL PEG. The MRSA was applied to marked regions of the pig’s skin with a sterile foam tip applicator. At T6, 24 hours after T5, final swabs were collected and tissue was harvested.

### Microbiome sequencing and analysis

#### DNA extraction

Bacterial DNA was extracted from swabs and stored at −80°C, as described previously (43). Swabs were incubated for 1 hour at 37°C with shaking in 300 µL yeast cell lysis solution (from Lucigen MasterPure Yeast DNA Purification kit) and 10,000 units of ReadyLyse Lysozyme solution (Lucigen). Samples were subjected to bead beating for 10 minutes at maximum speed on a vortex mixer with 0.5-mm glass beads (MoBio), followed by a 30-minute incubation at 65°C with shaking. Protein precipitation reagent (Lucigen) was added, and samples were spun at maximum speed. The supernatant was removed, mixed with isopropanol, and applied to a column from the PureLink Genomic DNA Mini Kit (Invitrogen). Instructions for the Invitrogen PureLink kit were followed exactly, and DNA was eluted in 50-µL elution buffer (Invitrogen). At each sampling event, swab control samples that never came into contact with the skin were collected, prepared, and sequenced exactly as the experimental samples. No significant background contamination from either reagent and/or collection procedures was recovered.

#### Sequencing and analysis

Amplification of the 16S rRNA gene V1–V3 region was performed as described previously (43). Sequencing was performed at the PennCHOP microbiome core on the Illumina MiSeq using 300-bp paired-end chemistry. The mock community control (MCC; obtained from BEI Resources, National Institute of Allergy and Infectious Diseases [NIAID], National Institutes of Health [NIH] as part of the Human Microbiome Project: Genomic DNA from Microbial Mock Community B [even, low concentration], v5.1L, for 16S rRNA Gene Sequencing, HM-782D) was sequenced in parallel. Sequencing of the V1–V3 region was performed using 300-bp paired-end chemistry. Sequences were pre-processed and quality-filtered prior to analysis, including size filtering to 460–600 nucleotides. HmmUFOtu was used for sequence alignment and phylogeny-based operational taxonomic unit clustering as described previously (44). Statistical analysis and visualization were performed using the phyloseq package (45) in the R statistical computing environment.

### Porcine bacteria isolation

Tubes containing skin swabs in PBS were vortexed vigorously for 10 minutes at room temperature (RT). Then 100 µL from each sample was spread on two blood agar (BA) plates. The initial swab samples (T1; Fig. 2A) were diluted 1:10 in PBS prior to plating. The BA plates were incubated at 37°C, 5% CO_2_ overnight, and RT for 3 days. Morphologically unique-looking colonies from each plate were picked and transferred to a second BA plate, which was incubated at 37°C overnight and used as the source plate for the screen below.

### Screen for MRSA-inhibiting isolates

Isolates from the BA source plates were inoculated into brain heart infusion medium containing 0.8% Tween 80 (BHI-T) in 96-well plates. After 24 hours at 37°C, cultures were resuspended and diluted 1:10 into fresh BHI-T. Following overnight incubation at 37°C, a 96-pin replicator was used to transfer 0.2 µL of each culture to blood agar (BA) plates on which 100 µL of an overnight culture of MRSA diluted to optical density at 600 nm (OD_600_) = 0.1 had been spread. Control plates without MRSA were also inoculated. The plates were incubated at 37°C for 24 hours, photographed, and colonies that inhibited the growth of MRSA, identified by a ring of no MRSA growth around the isolate, were selected.

### MALDI-TOF MS identification of MRSA-inhibiting porcine isolates

Isolates that exhibited the inhibition of growth of MRSA were streaked for single colonies on BA plates and incubated at 37°C overnight. Bacterial identification was performed using the MALDI Biotyper Microflex LT System (Bruker Daltonik GmbH) and the accompanying library, MBT BDAL 8468 MSP Library. According to the manufacturer’s instructions, a genus- and species-level identification is accepted with a MALDI-TOF MS score of ≥2.00 and a genus-level identification is accepted at a score of 1.75–1.99 when backed by other ancillary microbiological identification methods. Formic acid was applied to all Gram-positive organisms in the run to break down the cell wall. Full-tube extraction method was utilized for some sample identifications. All samples were run in duplicate.

### 16S rRNA gene Sanger sequencing

Colony PCR using GoTaq DNA polymerase (Promega) was performed on isolates not identified by MALDI-TOF MS. Primers (8F [AGAGTTTGATCCTGGCTCAG] and 1391R [GACGGGCGGTGTGTRCA]) (46) amplified the 16S rRNA gene, with an initial heating of 98°C for 3 minutes followed by 35 cycles of 95°C for 45 seconds, 50°C–58°C for 60 seconds, and 72°C for 90 seconds. PCR reactions were cleaned using Exo-CIP (NEB) and sequenced on an ABI 8730XL with BigDye Taq FS Terminator V3.1 (University of Pennsylvania, Penn Genomic Core). The DNA sequence was compared against the bacteria database of National Center for Biotechnology Information (NCBI) using the default settings of blastn.

### Construction of phylogenetic tree

Cultured bacterial isolates that could be definitively identified at the species level were included in the phylogenetic analysis. Representative 16S rRNA sequences for each species were curated from the Silva Living Tree Project (LTP) database (47). For species without an LTP entry, the longest high-quality sequence from the Reference Non-Redundant data set was used.

Multiple sequence alignment of representative 16S rRNA sequences was generated using MAFFT v. 7.505 using the L-INS-i setting (48). A maximum likelihood tree was constructed from the multiple sequence alignment with RAxML v. 8.2.12 using the best tree from 100 searches (49). The tree was midpoint rooted in FigTree v. 1.4.4 and visualized in RStudio. Pairwise cophenetic distances between species were calculated using the R ape package v 5.5.

### Bacteria–bacteria interaction screen

Eight MRSA-inhibiting isolates were tested against a collection of 88 pig isolates (37 of which could inhibit the growth of MRSA) in a growth inhibition spot diffusion assay. Single isolate colonies that had been grown on BA plates for 72 hours at RT were inoculated into TSB and incubated at RT. After 72 hours, the TSB cultures were diluted to an OD_600_ = 0.1 in PBS and 60 µL of the cell suspensions were spread on BA and BHI-T agar plates. The TSB cultures of the eight isolates to be spotted on the lawns were diluted to OD_600_ = 1.0 in PBS. For *D. incerta*, which grows to a low cell density, it was sometimes necessary to pellet the cells (10,000× *g*, 5 minutes at RT) and resuspend the cell pellet in a lower volume of PBS to achieve a cell suspension with an OD_600_ of 1.0. Five microliters of the cell suspensions were spotted on the bacteria lawns and control plates. The plates were incubated at RT for 72 hours and at 37°C for 24 hours, at which point they were photographed and zones of inhibition recorded.

Bacterial isolates that had not been identified to species level by either MALDI-TOF or 16S rRNA sequencing were excluded from the *in silico* analysis. For all growth conditions, each bacterial interaction was graded as inhibitory (1) or non-inhibitory (0) based on the presence of a visible zone of inhibition. Interactions across growth conditions were then consolidated such that a bacterial interaction was considered inhibitory if the inhibition was observed in at least one growth condition. A phylogenetic tree of all isolates was constructed from representative 16S rRNA sequences as described above. Pairwise cophenetic distances between species in the tree were calculated using the R ape package v 5.5.

### Whole-genome sequencing of bacterial genomes

Bacterial isolates from frozen stock were streaked on blood agar plates. A single bacterial colony was inoculated into 3 mL of tryptic soy broth (TSB) media and incubated at 37°C overnight without shaking. Genomic DNA was extracted using the ZymoResearch QuickDNA Fungal/Bacterial MicroPrep kit. Genomic DNA was sequenced by the PennCHOP Microbiome Core on the Illumina HiSeq2500.

### Genome assembly and annotation of biosynthetic gene clusters

Quality control of raw genomic reads was performed using FastQC v.0.11.8. The Nextera adapter sequence and 10 additional base pairs from each paired read were trimmed using TrimGalore v. 0.6.5. Quality control of trimmed reads was again performed using FastQC v.0.11.8. Genomes were assembled *de novo* with Unicycler v0.4.8 using standard settings. Whole-genome sequences of selected commensal bacteria were mined for BGCs with antiSMASH v 6.1.1 with all extra features enabled (29).

### Murine colonization experiments

All mouse procedures were performed under protocols approved by the University of Pennsylvania IACUC. Seven-week-old female SKH1-elite mice were purchased from Charles River (no. 477) and allowed to acclimate for 1 week before experimentation began. Mice were given *ad libitum* access to food and water. Pig commensal isolates were grown in liquid TSB at RT for 24 hours at 300 rpm, then 37°C for 24 hours at 300 rpm. On the following day, OD_600_ measurement was used to standardize inoculums, and pellets were resuspended in TSB to acquire 2 × 10^9^ CFU/mL inoculums. Titers were validated by culture and OD_600_ measurements. Mice were monoassociated at the dorsum by pipetting on 50 μL of pig isolate inoculum and then spread using a sterile swab. Applications of pig commensal suspensions were repeated 24 hours later. MRSA (ATCC no. BAA-1717; USA300 Rosenbach strain) was grown in TSB overnight at 37°C with 300 rpm shaking, with inoculum calculated and diluted similarly. A volume of 50 μL (of 2 × 10^9^ CFU/mL inoculum) was applied 24-hour post-association with the pig commensal isolate(s). All experiments were approved by the University of Pennylvania IACUC under protocol no. 805700.

### Statistical analysis and data visualization

All statistical analyses were performed using functions built into the R statistical environment (Rstudio version 1.4.1106). Data were visualized using ggplot2 (50) and ggtree (51). Log transformation was applied for all CFU count data before analysis. One-way ANOVA with Dunnett’s *post hoc* analysis was performed in Fig. 2D and E, Fig. 5B. Two-way ANOVA was performed in Fig. 2B, and Dunnett’s *post hoc* analysis using T1 group as control was performed on each treatment group separately. Bonferroni correction was applied to *P*-values from the *post hoc* analysis.

To further investigate the differences in group-level trajectories over time in Fig. 2B, we fitted the natural log values of the CFU counts using a random-effect GLS model with linear splines using Stata/MP 17 (StataCorp, College Station, TX, USA). A Wald test of equality of parameters between groups was used to determine statistically significant differences.

Normality of data was assessed by the Shapiro–Wilk test and visually by QQ-plot for the interaction data shown in Fig. 6. Wilcoxon test was used for data whose distribution departed significantly from normality (Fig. 6B and C) based on the Shapiro–Wilk test. Bonferroni correction for multiple hypothesis testing was applied where applicable (Fig. 6C).

## Data Availability

Raw reads for whole-genome sequences were submitted to NCBI GenBank and Sequence Read Archive, respectively, under BioProject accession no. PRJNA922076.

## SUPPLEMENTAL MATERIAL

Supplemental Material msphere.00177-23-s0001.pdf 8 Supplemental Figures and 1 Supplemental Table.

ASM does not own the copyrights to Supplemental Material that may be linked to, or accessed through, an article. The authors have granted ASM a non-exclusive, world-wide license to publish the Supplemental Material files. Please contact the corresponding author directly for reuse.
