# Supplementary material for: Harnessing diversity and antagonism within the pig skin microbiota to identify novel mediators of colonization resistance to methicillin-resistant Staphylococcus aureus
Source: mSphere. 2023 Jul 5;8(4):e00177-23. doi: 10.1128/msphere.00177-23 (PMC10449522; doi:10.1128/msphere.00177-23)
Supplement: Supplemental Material — 8 Supplemental Figures and 1 Supplemental Table. [file msphere.00177-23-s0001.pdf]

SUPPLEMENTAL MATERIAL

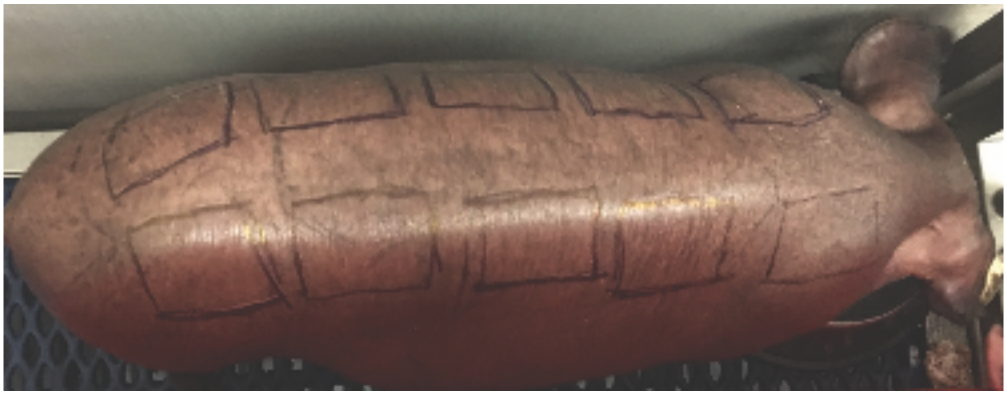

Figure S1: Example of pig model for topical treatment and colonization, with 10 patches marked with skin marker.

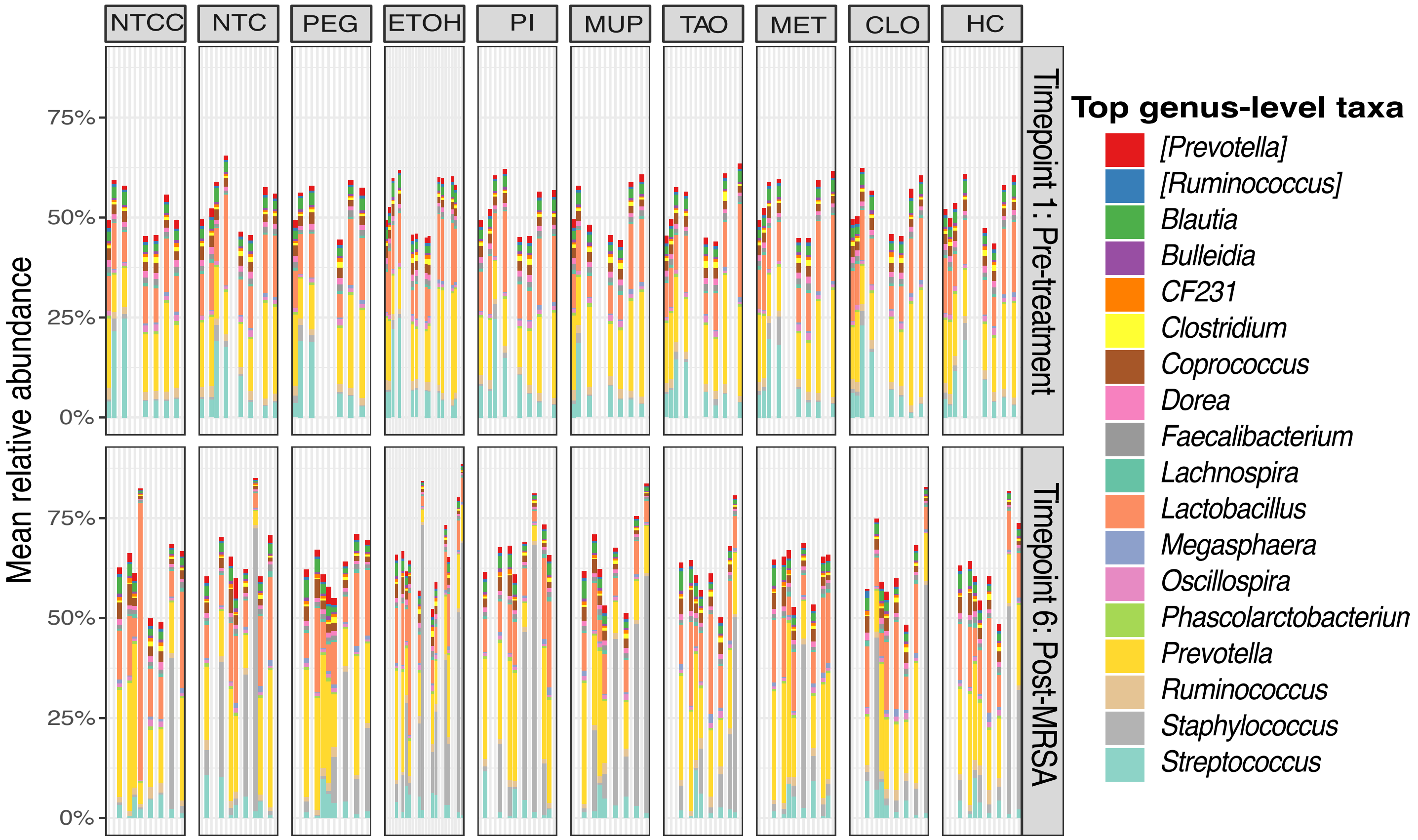

Supplemental Figure S2: Individual mean relative abundances (y-axis) of skin bacteria for each pig (x-axis) grouped by treatment. The top panel compares timepoint 1 before any treatments to the bottom panel at timepoint 6 following MRSA colonization. Empty columns represent swabs that either failed DNA extraction or did not yield sufficient sequencing reads to analyze.

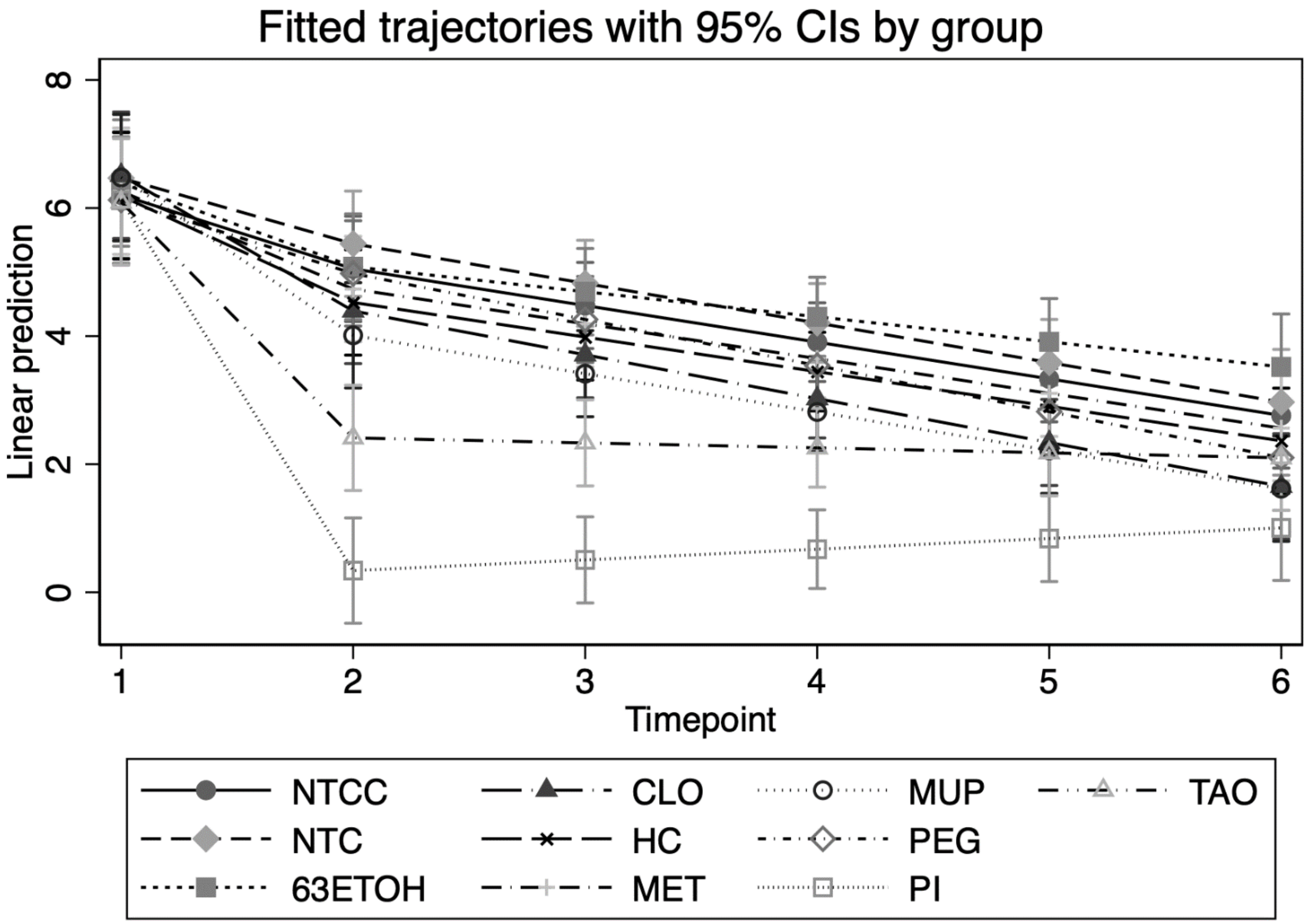

Supplemental Figure S3: GLS model with linear splines for data associated with Figure 2B (total bacterial CFU across time and treatment group).

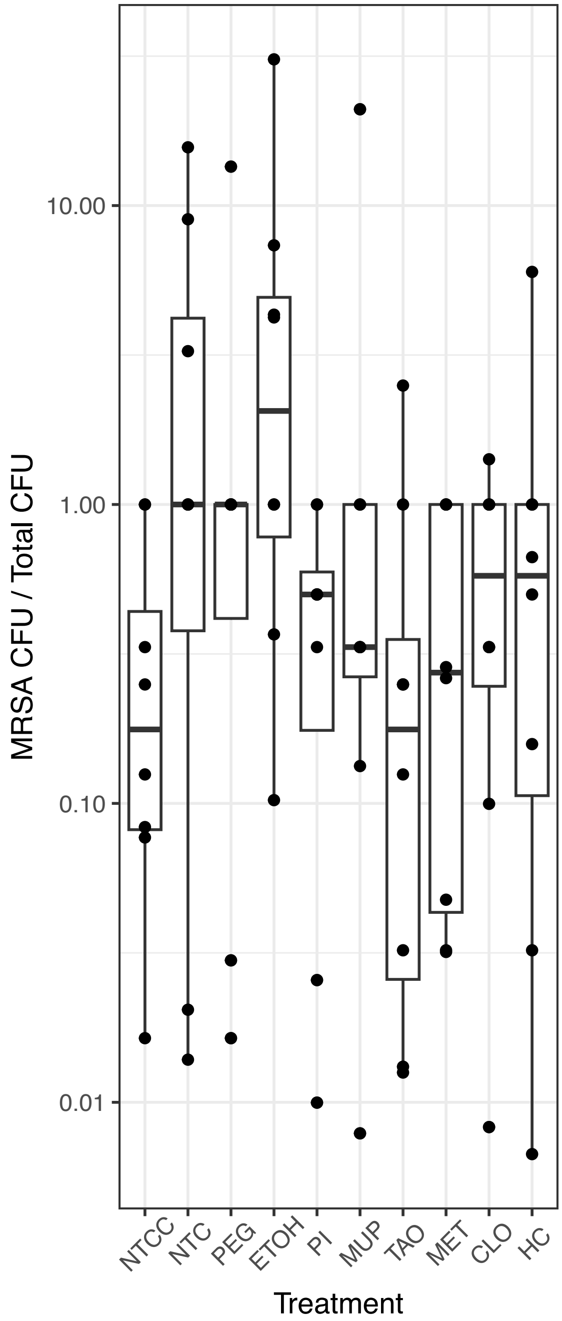

Supplemental Figure S4: Ratio of MRSA CFU to Total CFU recovered (Y-axis) at timepoint T6, according to treatment (x-axis).

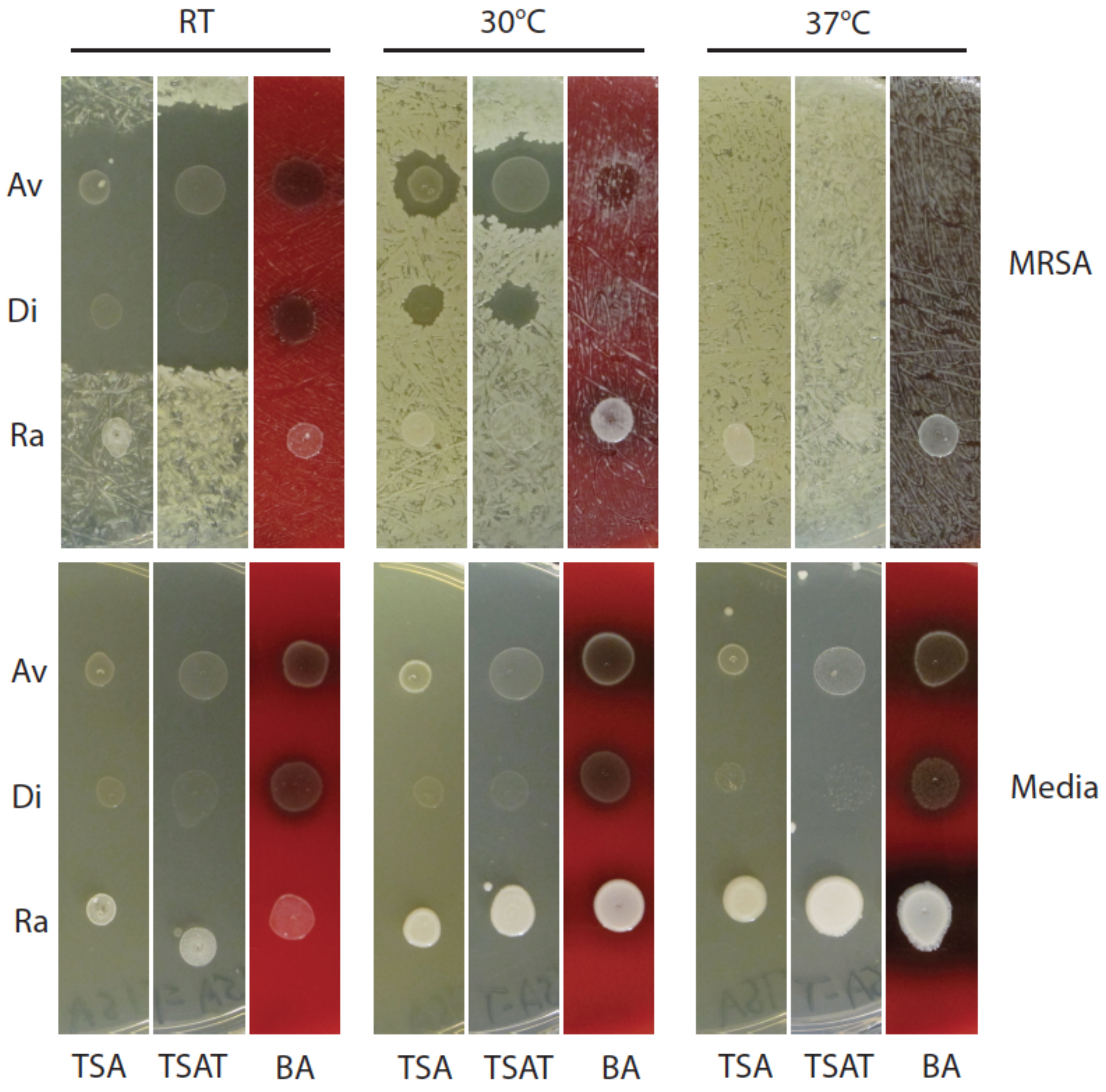

Supplemental Figure S5: Effect of temperature and media type of MRSA inhibition by *R. aerolata*, *A, viridans,* and *D. incerta*. MRSA lawns were prepared by spreading 60 ul of an OD600 = 0.1 suspension of overnight grown MRSA onto the respective media (TSA = Tryptic soy agar; TSAT = TSA with tween; BA = Blood agar). The lawn was dried for 1 hour at room temperature, then 5 ul of each pig isolate diluted to OD600 = 1.0 was spotted on top. Plates were incubated at the indicated temperatures for 42 hours prior to being photographed.

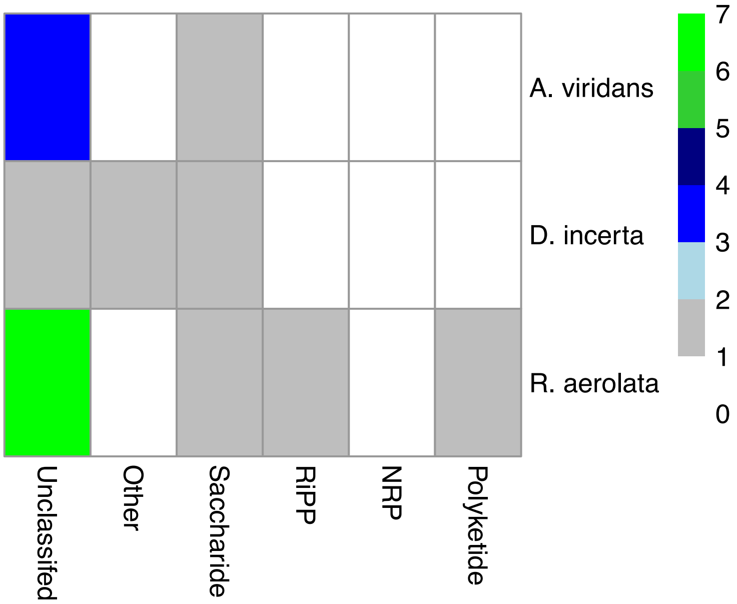

Supplemental Figure S6: Heatmap of biosynthetic gene clusters predicted by deepBGC. Colors represent number of BGCs in each class predicted for each species.

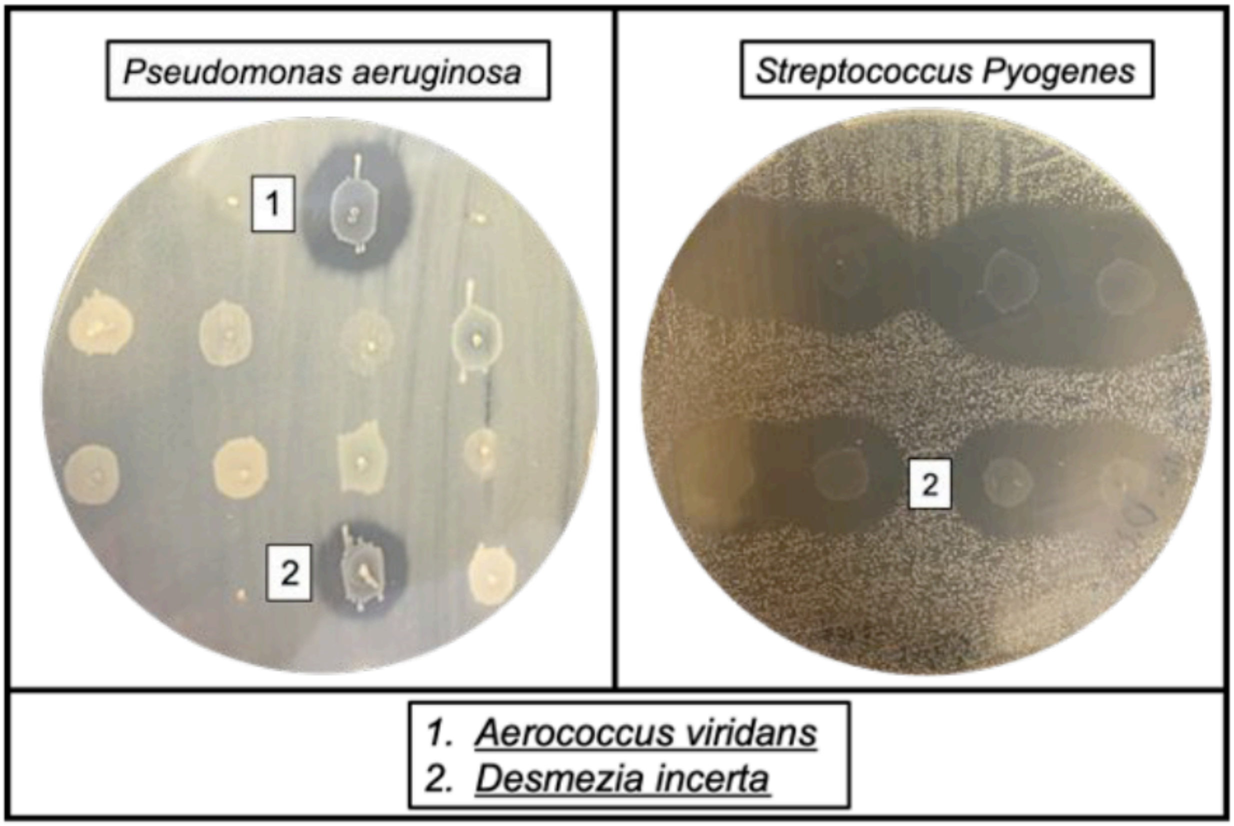

Supplemental Figure S7: Additional pathogens inhibited by *A. viridans* and *D. incerta*. A lawn of *Pseudomonas aeruginosa* (left) and *Streptococcus pyogenes* (right) were spotted with pig commensal bacteria. Zones of clearing marked with "1" indicate inhibition by *A. viridans* and "2" indicate *D. incerta*.

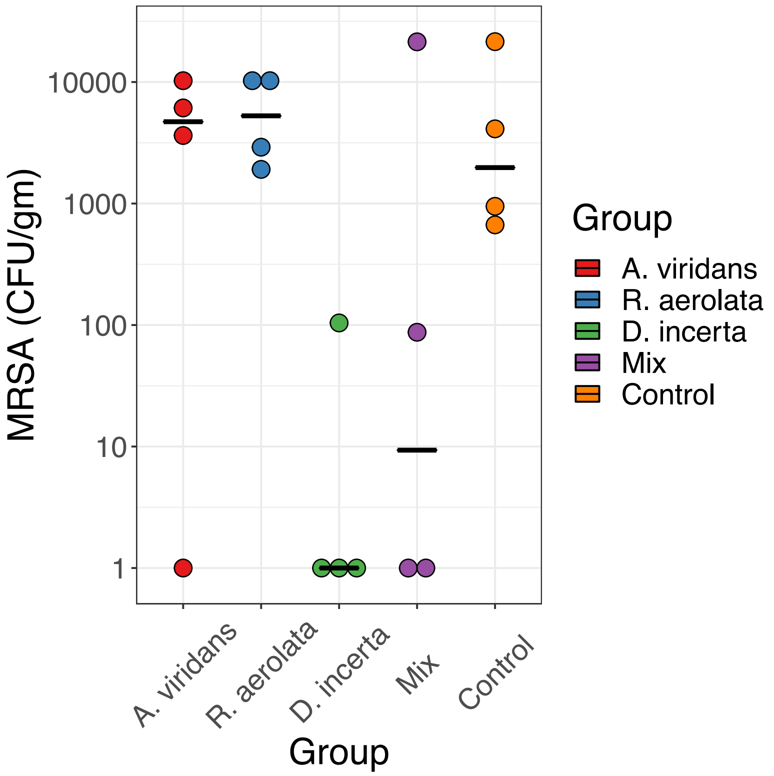

Supplemental Figure S8: Pilot experiment of pig isolates and in vivo colonization resistance to MRSA. Inoculum of $4.5x10^7$ CFU of each pig isolate was applied, daily for 2 days. On the 3rd day, MRSA inoculum of $1x10^8$ CFU was applied. Twenty-four hours later, skin was collected and CFU MRSA per gram tissue calculated (y-axis) for each treatment group (x-axis).

Supplemental Table S1: List of unique species isolated from skin of pigs

Isolate	Inhibits MRSA?
Acinetobacter lwoffii	Yes
Aerococcus suis	
Aerococcus viridans	Yes
Amycolatopsis alba	
Bacillus aerius	Yes
Bacillus altitudinis	Yes
Bacillus cereus or Bacillus mobiluss	Yes
Bacillus pumilis	Yes
Bacillus safensis	Yes
Bacillus sp or Kocuria carniphila	Yes
Bacillus subtilis	Yes
Bacillus tequilensis	Yes
Bacillus zhangzhouensis	Yes
Bergeyella porcine	
Brachyspira sp (muris?)	
Brevundimonas diminuta	Yes
Candida guilliermondii	Yes
Corynebacterium camporealensis	
Corynebacterium confusum	
Corynebacterium sp	
Corynebacterium lipophiloflavum	
Corynebacterium mycetoides	
Corynebacterium pollutisoli	
Corynebacterium glutamicum	
Corynebacterium sphenisci	
Corynebacterium spheniscorum	
Corynebacterium tuscaniense	
Corynebacterium vitaeruminis	
Corynebacterium xerosis	
Cryptococcus magnus	Yes
Delftia acidovorans	Yes
Desemzia incerta	Yes
Enterococcus casseliflavus	Yes
Enterococcus faecalis	Yes
Enterococcus saccharolyticus	
Escherichia vulneris	Yes
Facklamia hommis	
Kocuria atrinae	
Kocuria gwangalliensis	
Kocuria rhizolphilla	Yes
Leucobacter chromiiresistens	
Lysinibacillus fusiformis	Yes
Microbacterium arborescens	Yes
Microbacterium liquefaciens	Yes
Microbacterium maritypicum	

Microbacterium oxydans	
Microbaterium paraoxydans	Yes
Micrococcus endophyticus	
Moraxella osloensis	
Moraxella pluranimalium	
Neisseria perflavia	
Paenibacillus sIlvae	Yes
Pseudomonas azotoformans	
Pseudomonas chlororaphis	
Pseudomonas cremoricolorata	
Pseudomonas fulva	Yes
Pseudomonas koreensis	Yes
Pseudomonas oryzihabitans	
Pseudomonas parafulva	
Rhodococcus erthropolis	
Roseomonas gilardii	
Rothia aerolata	Yes
Rothia nasisurum	Yes
Staphylococcus chromogenes	Yes
Staphylococcus cohnii	
Staphylococcus divriesel	
Staphylococcus equorum	Yes
Staphylococcus gallinarum	
Staphylococcus haemolyticus	Yes
Staphylococcus homis	
Staphylococcus petrasii	
Staphylococcus sciuri	
Staphylococcus simulans	Yes
Staphylococcus sp	
Staphylococcus sp presumptive xylosus	Yes
Staphylococcus warneri	Yes
Stenotrophomonas maltophilia	Yes
Streptococcus alactolyticus	
Streptococcus gallolyticus	
Streptococcus oralis	
Streptococcus porcorum	
Streptococcus suis	
Streptococcus tangierensis	
Trichosporon asahii	Yes
Isolates were identified by MALDI-TOF Mass spectrometry and 16S rRNA gene sequence	
Isolates (37) that inhibited MRSA were preferentially collected.	
